# Tea disease identification based on ECA attention mechanism ResNet50 network

**DOI:** 10.3389/fpls.2025.1489655

**Published:** 2025-02-06

**Authors:** Lanting Li, Yingding Zhao

**Affiliations:** School of Software, Jiangxi Agricultural University, Nanchang, China

**Keywords:** tea plant diseases, ECA attention mechanism, ResNet50, deep learning, leave

## Abstract

Addressing the challenge of identifying tea plant diseases against the complex background of tea gardens, this study proposes the ECA-ResNet50 model. By optimizing the ResNet50 architecture, adopting a multi-layer small convolution kernel strategy to enhance feature extraction capabilities, and introducing the ECA attention mechanism to focus on key features, the model achieves a 93.06% accuracy rate in tea disease identification, representing a 3.18% improvement over the original model, demonstrating industry-leading performance advantages. This model not only accurately identifies tea diseases in gardens but also possesses excellent generalization capabilities, performing outstandingly on datasets of other plant categories. These results indicate that ECA-ResNet50 can effectively mitigate the interference of complex backgrounds and precisely recognize tea disease targets.

## 1 Introduction

The tea industry in China has undergone years of development and continues to grow steadily, occupying an important position in the domestic market and enjoying a strong reputation internationally. However, throughout the cultivation process, tea plants inevitably face various diseases and pests, which not only severely affect tea yields but also pose a serious threat to the quality of the tea. To effectively address this challenge, it is essential to actively introduce and apply emerging technologies such as artificial intelligence, enabling precise and rapid detection and effective control of tea diseases, thereby ensuring the sustainable and healthy development of the tea industry.

Computer vision, as an important branch of artificial intelligence technology, aims to enable machines to possess visual perception capabilities similar to those of humans (Yu et al., 2023). Currently, many countries are actively exploring the practical applications of computer vision in the agricultural sector, achieving significant research results. Among these, employing deep learning technology for crop disease recognition, followed by the application of effective control strategies, has emerged as a pivotal trend shaping agricultural progress. The application of this technology not only allows computers to provide rapid and accurate diagnostic results but also significantly enhances the quality and overall yield of crops while reducing additional labor costs and time consumption, thereby providing strong support for the sustainable development of agriculture (Dhanya et al., 2022).

In 2016, Li (2017) designed a tobacco disease diagnosis system based on a six-layer convolutional neural network model. This system utilizes deep learning techniques to identify tobacco diseases and provides convenient diagnosis and prevention services for growers through web design. The research further delved into the impacts of varying iteration numbers and resolutions on the training efficiency and classification capabilities of the network model. In 2017, Sun et al. (2017) and his team introduced a convolutional neural network framework incorporating batch normalization and global pooling methodologies. After adjusting the network structure and parameters, this model greatly improved the accuracy, efficiency, and stability of plant disease identification. Optimizations resulted in the best model significantly outpacing traditional convolutional neural networks in convergence speed, achieving an accuracy rate exceeding 90% after just three training iterations. Furthermore, this proposed model necessitates minimal computational demands, featuring a parameter memory of merely 2.6 MB, and attained a remarkable average testing recognition accuracy of 99.56%, with comprehensive performance for recall and precision reaching 99.41%. These improvements enable the model to deliver efficient and accurate performance in the field of plant disease identification. In 2018, Lu et al. (2018) and colleagues proposed a deep learning-based recognition method for rice leaf disease images. They constructed a rice disease image database and employed PCA (Principal Component Analysis) for dimensionality reduction. Utilizing the Caffe deep learning framework, they crafted a profound network architecture encompassing four convolutional tiers, three pooling stages, and a solitary fully connected layer. Training and simulation with 2,000 rice disease images, combined with ten-fold cross-validation testing, verified that the designed deep learning structure and learning algorithm achieved an average recognition rate of 96.9% for common diseases such as rice blast and sheath blight in northern cold region rice. The experimental results thoroughly demonstrated the effectiveness of this method in identifying major rice leaf diseases, providing strong technical support for accurate recognition and prevention of rice diseases. In 2019, Wu (2019) proposed a tomato leaf disease recognition technology based on a deep residual network. This technology automatically adjusts the key hyperparameters in the network using a Bayesian optimization algorithm, streamlining the training procedure for the deep learning network. By incorporating residual units into the traditional neural network structure, it mitigated potential concerns related to gradient vanishing and explosion phenomena within deep networks significantly enhancing the performance of the network model and allowing for precise extraction of high-dimensional features from tomato leaf images. These features were then used for accurate disease identification. Experiments showed that the deep residual network model in this study achieved recognition accuracy exceeding 95% for common tomato leaf diseases such as powdery mildew, early blight, late blight, and leaf mold on public datasets. This study offers a noteworthy reference for swiftly and precisely identifying tomato leaf diseases. In 2020, Ji et al. (2020) and colleagues adopted a convolutional neural network based on an improved residual network, using publicly available plant image datasets for training. Comparative experiments with the Xception and VGG-16 network models showed that the improved neural network model achieved an accuracy rate of 98.6%, significantly surpassing Xception’s 93% and VGG-16’s 95%, demonstrating its efficiency and accuracy. In 2021, Wang et al. (2021) and colleagues proposed an improved CenterNet-SPP model for potato leaf diseases. This model first precisely locates the central points of the targets using a feature extraction network, and then accurately obtains key image information such as center point offset and target size through center point regression techniques. The experiments demonstrated that the model attained a mean average precision (mAP) score of 90.03% on the validation dataset. In 2022, Sun and Lin (2022) and colleagues introduced a novel approach for detecting apple leaf diseases, leveraging ensemble learning techniques. This method integrates the YOLOv5 and EfficientDet models, achieving model integration through a non-maximum suppression algorithm. Testing showed that the new method effectively improved the detection performance of three common apple leaf diseases without sacrificing detection speed, with average precision rising to 73.4%. Compared to the individual use of YOLOv5 and EfficientDet, the new method improved accuracy by 3.0% and 4.8%, respectively. In 2023, Li et al. (2023) and colleagues constructed an alfalfa disease recognition model using an improved AlexNet deep learning convolutional neural network, trained on a dataset of 13 common alfalfa diseases. After comparing different image input resolutions, they found that the optimal model achieved the highest recognition accuracy with an input size of 512 pixels × 512 pixels, reaching an overall recognition accuracy of 72%. After further excluding low-accuracy samples, the recognition accuracy for five key alfalfa diseases significantly increased to 92%. In 2024, Qiu et al. (2024) and colleagues developed an algorithm called CBAM-YOLOv5l based on an improved YOLOv5. Through experiments, they confirmed that the method enhanced detection accuracy without compromising on the swiftness of the detection process. The algorithm achieved an overall average precision of 96.52% on the validation set, with an average detection time of 27.52 ms, demonstrating significant advantages in detection accuracy compared to other object detection algorithms like YOLOv4, YOLOv4-Tiny, and Faster R-CNN.

Currently, investigations into recognizing plant leaf diseases and pests with convolutional neural networks predominantly depend on conventional frameworks devoid of an attention weighting mechanism. This can lead to a misalignment of the model’s focus, subsequently affecting recognition accuracy. Moreover, the aforementioned studies have not applied the improved models to the recognition of diseases and pests in other crop leaves, making it impossible to comprehensively validate their generalization capabilities. To tackle these challenges, this research introduces the ECA-ResNet50 model, which integrates the ECA attention mechanism with the ResNet50 network framework. This model focuses on various tea leaf diseases, such as algal leaf disease, anthracnose, and bird’s eye spot disease, as well as healthy tea leaves. Through comparative experiments with traditional convolutional neural networks, the effectiveness of ResNet-ECA in tea disease recognition was validated. Additionally, to further assess the generalization performance of the improved model, it was applied to train and validate datasets of disease and pest leaves from other crops, including corn, apples, and potatoes.

## 2 Research and implementation of algorithm

### 2.1 Dataset construction

#### 2.1.1 Data acquisition for dataset

**Figure 1:**
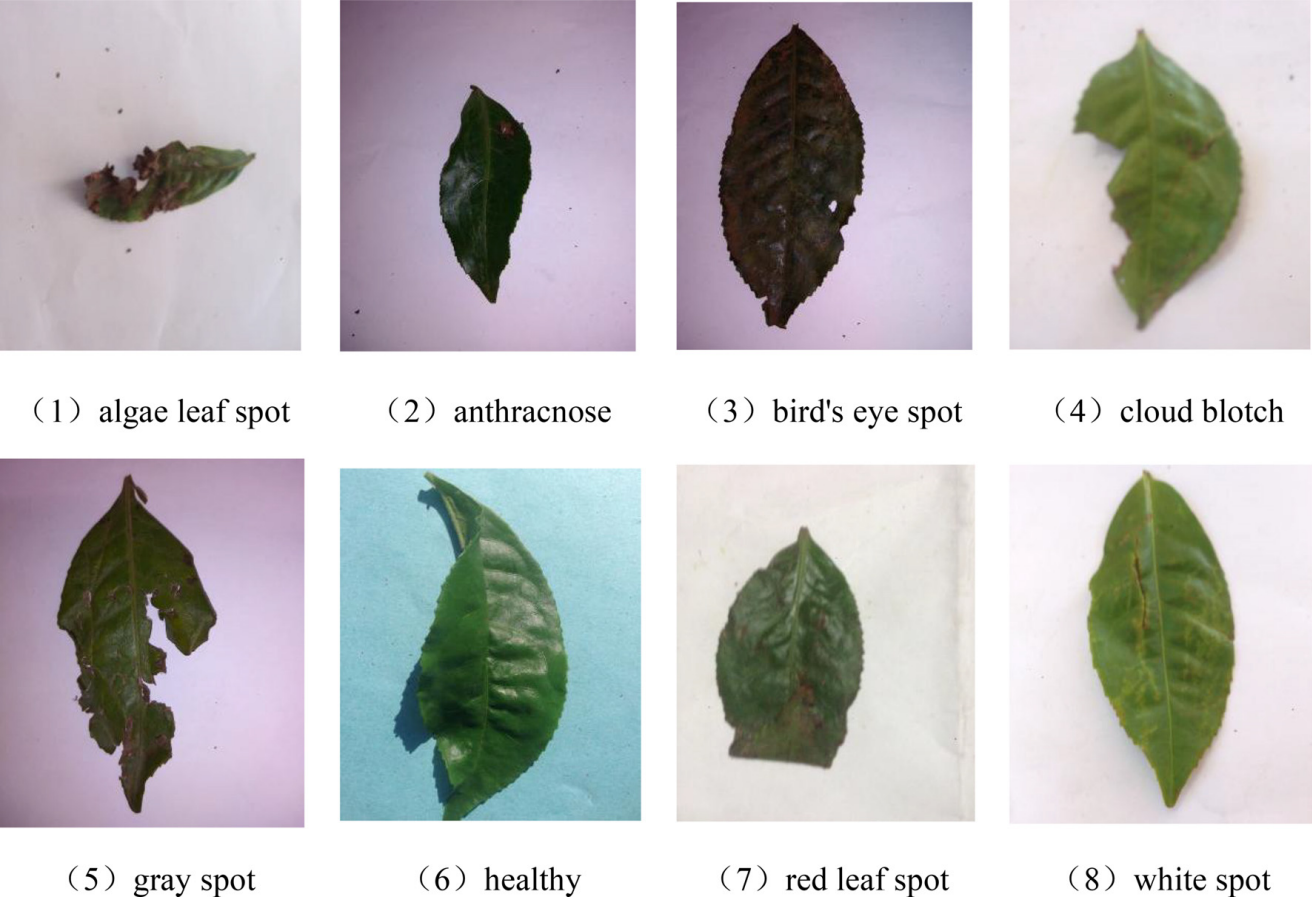
Images of tea diseases.

According to statistical data analysis of the system, China’s tea plants suffer from a wide variety of diseases, totaling approximately over 140 types, which are widely distributed across various parts of the tea plants, including leaves, stems, roots, and flowers (Chen, 2022). Given the limitations of experimental conditions, this study collected a total of 885 images of tea diseases through search engines (https://www.kaggle.com/datasets/shashwatwork/identifying-disease-in-tea-leafs). After meticulous identification and classification by authoritative experts, these images were categorized into seven distinct types of leaf diseases, as well as healthy leaves. The seven disease types are algae leaf spot, anthracnose, bird’s eye spot, cloud blotch, gray spot, red leaf spot, and white spot disease. Some images of tea disease leaves are shown in **Figure 1**.

#### 2.1.2 Dataset processing

**Figure 2:**
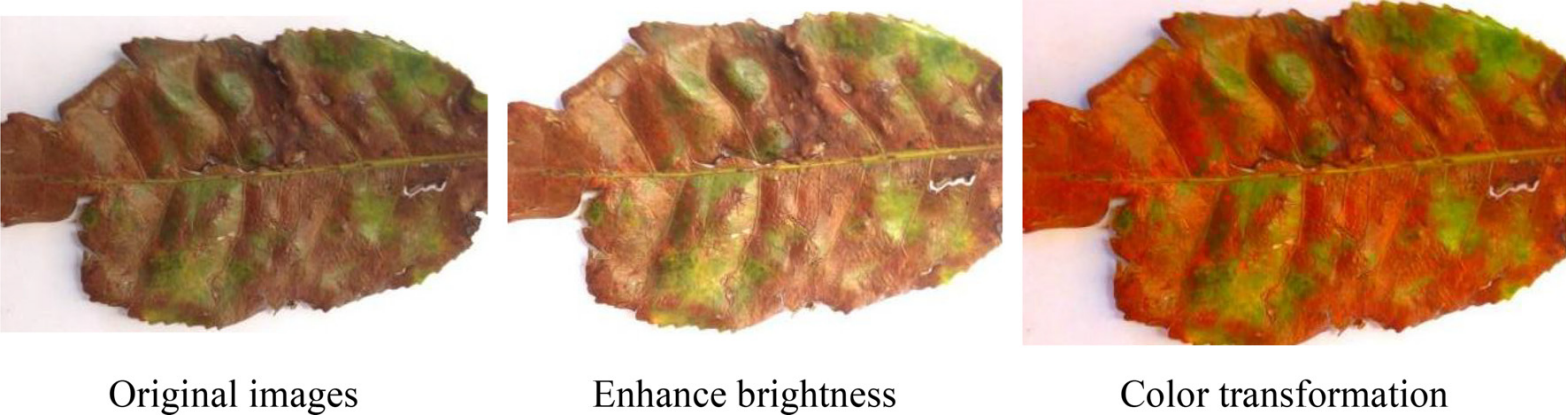
Image enhancement examples.

During the training phase of a Convolutional Neural Network (CNN) model, ensuring a large-scale and diverse dataset plays a decisive role in enhancing the model’s performance. However, acquiring a sufficient number of images that cover various types of tea plant disease under current conditions is a formidable challenge. To address this issue, this research employs data augmentation strategies to efficiently augment the training dataset thereby improving the model’s generalization capability and recognition accuracy for tea plant disease images. Firstly, the original dataset is expanded through a series of data augmentation techniques, including flipping, rotation, cropping, color transformation, and blurring, with each method expanding the dataset to 1000 images. Some examples of the augmented images are shown in **Figure 2**. Subsequently, the expanded dataset is divided into a training set and a test set at an 8:2 ratio. During the data preprocessing stage, to ensure data consistency and compatibility with the model’s input requirements, all images are resized to a uniform dimension of 224×224 pixels. Furthermore, through padding and random shuffling, we aim to fully utilize the data information and enhance the model’s training effectiveness.

### 2.2 ECA mechanism

**Figure 3:**
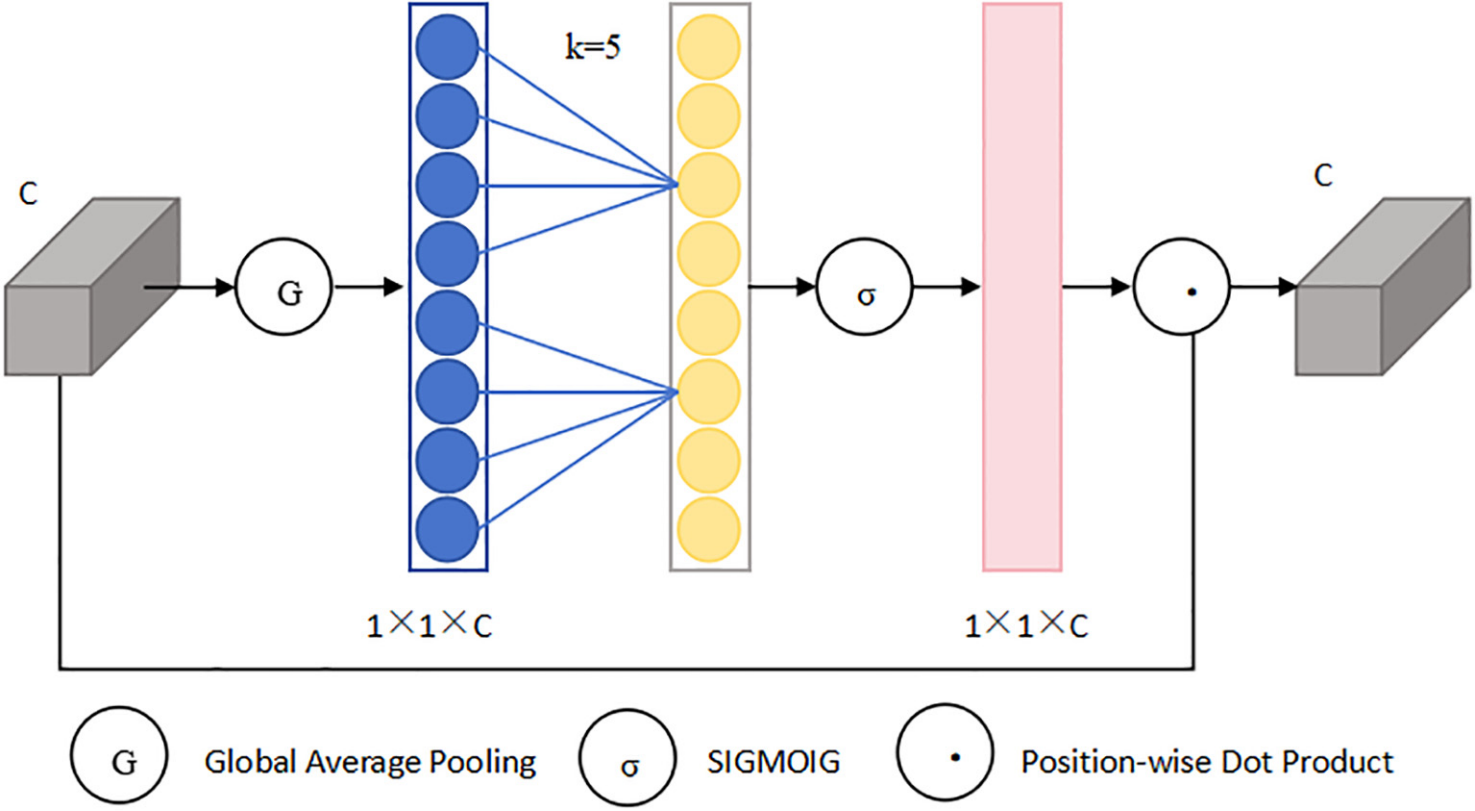
ECA architecture diagram.


The ECA (Wang et al., 2020) module is an optimized version of the SE (Hu et al., 2019) attention module that significantly enhances performance despite having fewer parameters. When performing global average pooling, it ingeniously avoids compressing the channels of the input feature map, aiming to mitigate the adverse effects of learning inter-channel dependencies. Within the ECA module, the extent of local cross-channel interaction is defined as k, meaning each channel and its adjacent k channels are considered. By utilizing a one-dimensional fast convolution tailored to the k value, the module efficiently accomplishes local cross-channel interaction, capturing the relationships among channels. Finally, the weights, post-processed via a Sigmoid function, are scaled with the corresponding entries in the input feature map to yield the output. Its structural diagram is shown in **Figure 3**. The distinctive architecture of the ECA module enables the model to prioritize the feature information pertaining to smaller objects, ensuring both efficiency and computational effectiveness. Since the k value is proportional to the number of channels, to avoid cross-validation, the k value can be obtained through Equation 1:

(1) $$K={\left|\frac{1bC+b}{\gamma }\right|}_{odd}$$

In the equation, C denotes the channel count in the input feature map, while b and γ are conventionally initialized as 1 and 2, respectively, respectively, to adjust the ratio between the dimensions of the convolutional kernel and the value of C. The notation odd indicates that K should be the odd number closest to the function’s value.

### 2.3 ResNet50

ResNet50 (He et al., 2016) is a deep convolutional neural network-based algorithm designed for image classification tasks, proposed by Kaiming He and his colleagues at Microsoft Research in 2015. As an important member of the ResNet family, ResNet50 addresses the issue of gradient vanishing during the training of deep networks by introducing residual connections, effectively enhancing the model’s performance.

**Figure 4:**
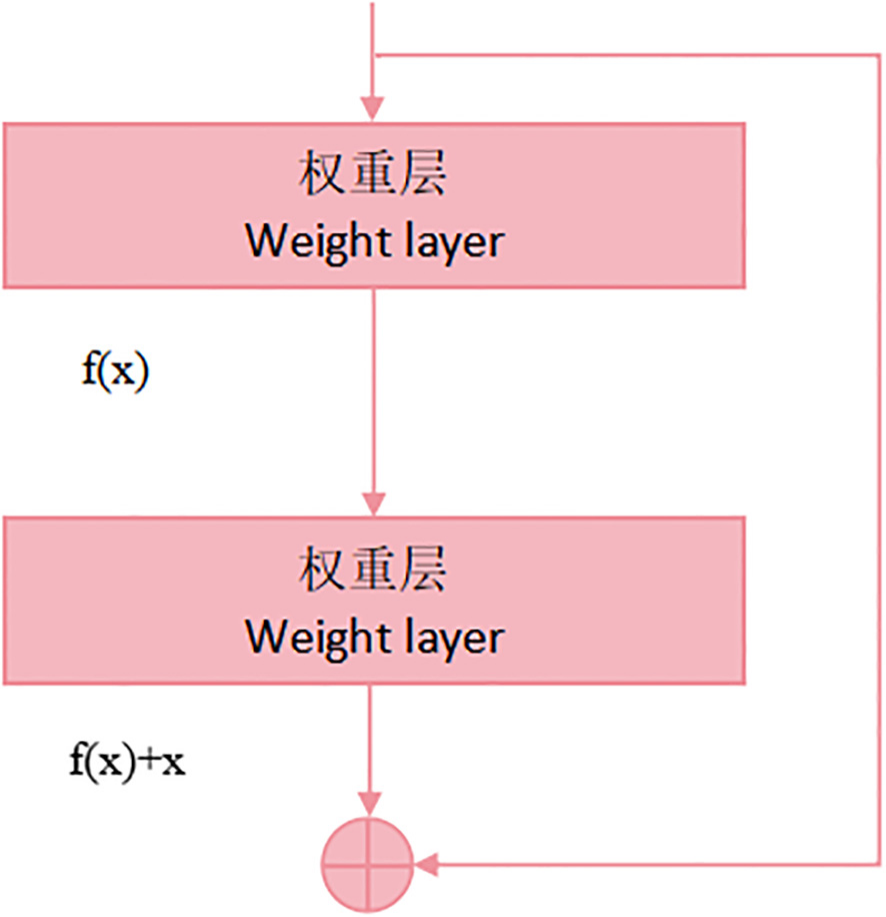
Residual block structure diagram.

The ResNet50 architecture comprises numerous residual blocks, which include additional layers such as pooling layers and fully connected layers. The overall structure of the network is very deep, employing 50 convolutional layers, hence the name ResNet50. These convolutional layers extract features from images at different sizes and depths, enabling the model to capture features at various levels. The configuration of a residual block is depicted in **Figure 4**.

Each residual block is linked to each other by residual connections. This direct connection mitigates the issue of vanishing gradients by enabling the seamless flow of information across network layers. In ResNet50, each residual block consists of two convolutional layers, called the main path and the hop connection, respectively. By adding the input to the output of the main path, the residual learning of the information is realized. The formula for each residual element is as follows:


(2) $$x_{j+1}=x_{j}+F(x_{j},W_{j})$$

where $x_{j}$ 、 $x_{j+1}$ denotes both the input and output information of the layer network, respectively, and represents the learnable parameters within that layer. Perform a recursive operation on Equation 2 to obtain the relational expression of any deep J and shallow J:


(3) $$x_{J}=x_{j}+\sum_{i=j}^{J-1}F(x_{i},W_{i})$$

According to the chain derivative used in the backpropagation algorithm, the gradient of backpropagation can be expressed as:


(4) $$\frac{\partial \epsilon }{\partial x_{j}}=\frac{\partial \epsilon }{\partial x_{J}}\frac{\partial \epsilon }{\partial x_{J}}=\frac{\partial \epsilon }{\partial x_{J}}\left[1+\frac{\partial }{\partial x_{j}}\sum_{i=j}F(x_{i,}w_{i})\right]$$

Because all $\frac{\partial }{\partial x_{j}}\sum_{i=j}F(x_{i},W_{i})$ in Equation 4 may be equal to −1, this unit effectively mitigates the issue of information loss during the learning phase.

### 2.4 Network architecture based on ECA attention mechanism and ResNet50

**Figure 5:**
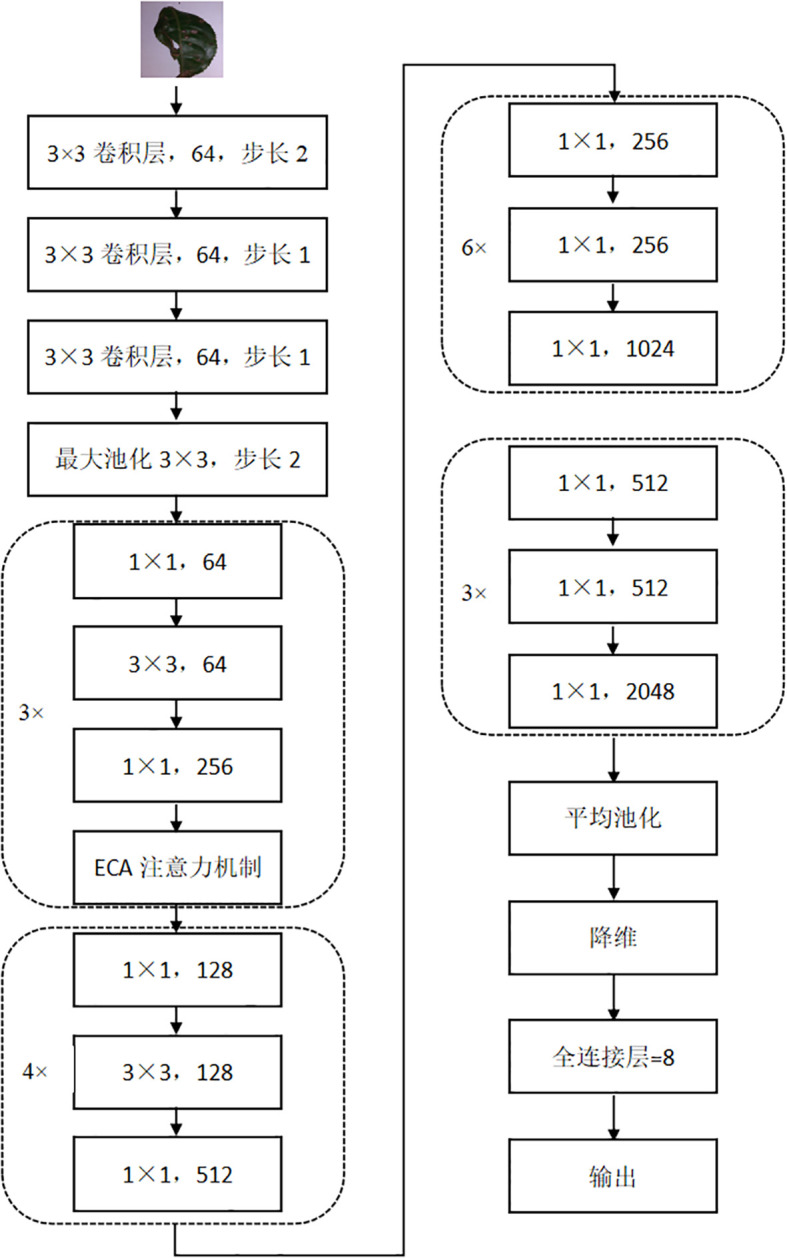
Structure diagram of ECA-ResNet50.

The ECA-ResNet50 model is optimized and improved on top of the ResNet-50 infrastructure. Firstly, the 7×7 convolution kernel of the first layer of ResNet-50 was replaced by three 3×3 convolution kernels. In the traditional ResNet50, the 7×7 convolution kernel is designed to capture a wider range of spatial context information in the input image, however, in the tea disease identification scenario, the disease characteristics are often complex and subtle, and the affected area is comparatively minute. In view of this, the strategy of using multi-layer small convolutional kernel not only refines the granularity of feature extraction and improves the accuracy of disease identification, but also enhances the learning ability and complexity of the model by reducing the total number of parameters and increasing the network depth, and significantly optimizes the performance. Moreover, to enhance the model’s sensitivity and recognition efficiency towards tea disease characteristics even further, ECA-ResNet50 integrates the ECA attention mechanism into the first residual module of ResNet-50. Although ResNet-50 itself can effectively alleviate the gradient problem in deep network training, relying solely on numerical transfer may not be enough to accurately capture the key features when dealing with tea diseases with similar characteristics, which will affect the recognition accuracy and generalization ability. By introducing the ECA attention mechanism, the model can focus on more discriminative feature information in the image, which effectively enhances the learning and recognition ability of tea disease characteristics, which is a key measure to improve the overall performance of the model, **Figure 5** is the structure diagram of ECA-ResNet50.

### 2.5 Experimental parameters and evaluation metrics

Precision, recall, accuracy, and F1-score were employed to assess the network model’s performance in identifying tea diseases. The formulas for calculating these evaluation metrics are outlined below:


(5) $$precision=\frac{TP}{TP+FP}$$

(6) $$recall=\frac{TP}{TP+FN}$$

(7) $$accuray=\frac{TP+TN}{TP+FN+FP+TN}$$

(8) $$F1=\frac{2TP}{2TP+FP+FN}$$

Here, TP denotes the count of samples accurately labeled as positive by the model, TN represents the count of samples correctly identified as negative. FP signifies the number of negative samples mistakenly predicted as positive, while FN represents the number of positive samples incorrectly labeled as negative. Accuracy gauges the fraction of samples correctly predicted by the model among all test samples, calculated as the total number of correctly predicted samples divided by the total test samples. Precision focuses on the ratio of samples predicted as positive by the model that are actually positive, computed as the number of correctly predicted positive samples divided by the total number of samples predicted as positive. Recall, also known as the true positive rate, assesses the proportion of actual positive samples accurately identified by the model, calculated as the number of correctly predicted positive samples divided by the total number of positive samples. F1 score serves as a comprehensive metric, balancing the significance of precision and recall by computing their harmonic mean. A higher F1 score signifies superior performance in both precision and recall, making it a frequently utilized evaluation metric for classification models.

## 3 Results and discussion

### 3.1 Experimental environment

This investigation is conducted utilizing the TensorFlow platform of the Python programming language, encompassing two distinct phases: model training and testing. In terms of hardware environment, IT uses an intel(R) Xeon(R) Silver 4112 processor with a frequency of 2.6 GHz. 16 GB of memory space; NVIDIA Quadro RTX5000 graphics card. In terms of software environment configuration, CUDAToolkit 10.0, CUDNN 10.1 and TensorFlow 2.2 are selected as the deep learning framework, and the operating system is Windows 10.

### 3.2 Activation function comparison experiment

In neural network models, activation functions play a very important role, which greatly enhances the network’s ability to process complex data and function mapping by giving the network nonlinear ability, adjusting the output range, and promoting sparse expression (Bahdanau et al., 2014). To enhance the efficiency and effectiveness of the model, optimization efforts are undertaken, three activation functions, ReLU (Xu et al., 2016), LeakyReLU (Technicolor T. et al., 2019) and ELU, were selected for training and comparison, so as to select the activation function strategy that is most consistent with the model. The experimental results are shown in **Table 1**, as evident from the tabular data, the ReLU activation function exhibits favorable performance in terms of accuracy, recall, and F1-score, and its accuracy is 1.68% and 7.5% higher than that of LeakyReLU and ELU, respectively. The above data show the superiority and applicability of the ReLU activation function in the ECA-ResNet50 model adopted in this study, and it can give full play to the potential of the model and achieve better performance than the other two activation functions.

**Table 1:** Activation function comparison experiment.

Activate the function	The number of iterations	Precision%	Recall%	F1%	Accuracy%
ReLU	200	93.09	93.06	93.07	93.06
LeakyReLu	200	91.43	91.38	91.40	91.38
ELu	200	87.71	85.56	86.62	85.56

### 3.3 Comparative experiments on attention mechanisms

Within the framework of neural network designs, the attention mechanism module plays a pivotal role, as an additional component of the neural network, can selectively focus on a specific part of the input, or effectively filter the information by assigning differentiated weights to different elements of the input. In recent times, due to its substantial contribution to enhancing model performance, this mechanism has garnered widespread adoption and implementation across diverse fields. In this study, three mainstream attention mechanisms, ECA, SE, and CABM (Fe et al., 2017), were selected to test and evaluate their respective effects in enhancing model performance. **Table 2** shows the performance comparison results achieved after introducing these three attention mechanisms into the model. Based on an examination of the experimental data, under the same experimental environment settings and conditions, the ECA attention mechanism has the best effect among the three attention mechanisms, showing the best performance, with an accuracy of 93.06%, exhibiting a 3.5% increase in comparison to the SE attention mechanism within the model and 1.81% more accurate than the CBAM attention mechanism. These results show that the ECA attention mechanism can more effectively enhance the recognition ability and robustness of the model in this experimental model.

**Table 2:** Comparative experiments on attention mechanisms.

Attention mechanisms	Batch size	The number of iterations	Activate the function	Accuracy%	#P
Join ECA	64	200	ReLu	93.06	23,569,869
Join SE	64	200	ReLu	90.56	23,585,736
Join CBAM	64	200	ReLu	91.25	23,585,834

### 3.4 Ablation experiments

**Figure 6:**
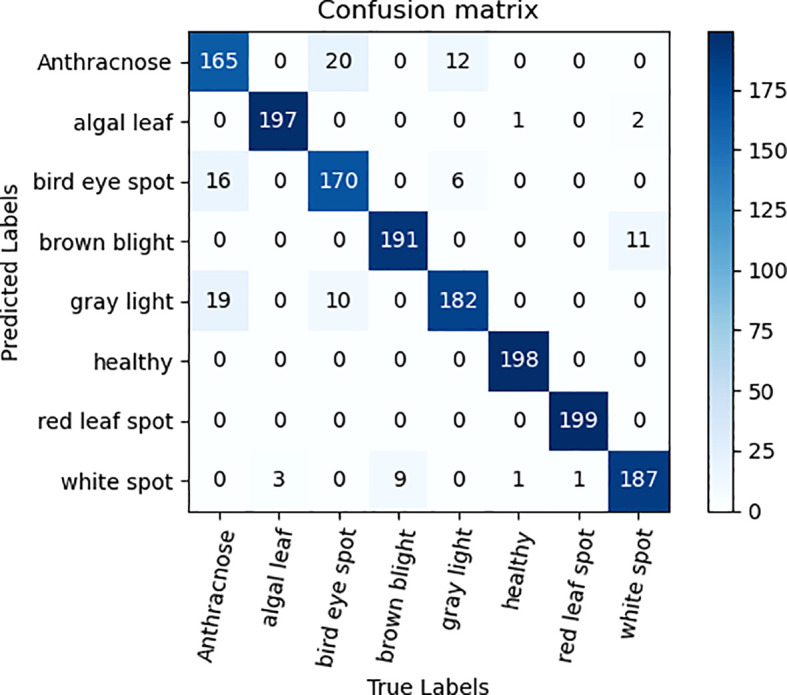
Confusion matrix diagram of ECA-ResNet50.

**Figure 7:**
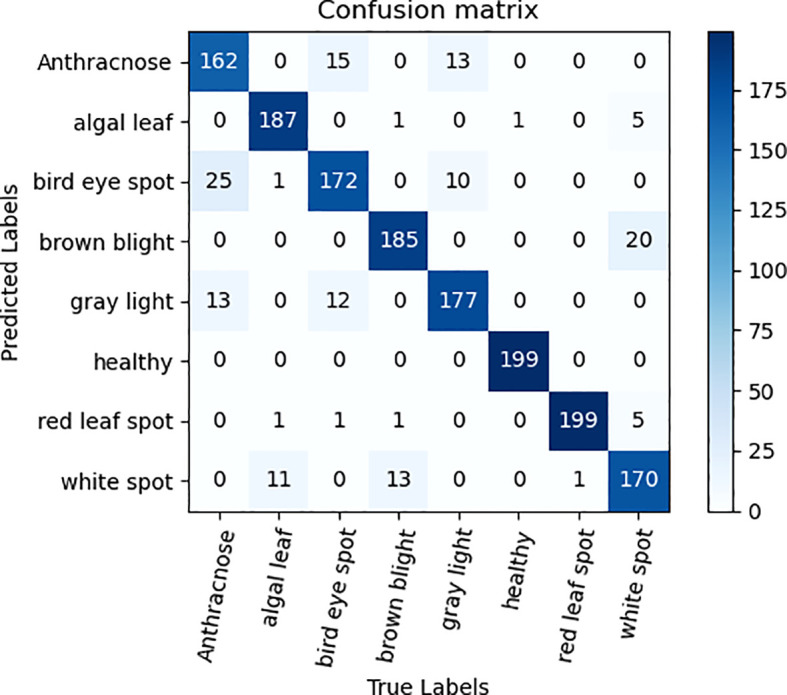
Confusion matrix diagram of ECA-ResNet50.

To validate the efficacy of the ECA attention mechanism module alongside three 3×3 convolutional kernel modules, ablation experiments were performed on the tea dataset, utilizing ResNet50 as the foundation network. The qualitative comparative outcomes are presented in **Table 3**. As can be seen from the data analysis in **Table 3**, the recognition accuracy of the model is significantly improved by 1.82% after the ECA attention mechanism is integrated into the ResNet50 model. The notable enhancement stems from the integration of the attention mechanism, empowering the model to precisely concentrate on the pivotal distinguishing characteristics within the image, thereby enhancing the recognition and learning efficiency of tea disease features, and improving the overall performance of the model. In addition, the replacement of three 3×3 convolution kernels with one 7×7 convolution kernel also brings a slight improvement in recognition accuracy. This improvement is due to the refinement of feature extraction brought about by the multi-layer small convolutional kernel design, which not only reduces the total number of model parameters, additionally, it enhances the intricacy and learning capacity of the model by augmenting the depth of the network, thereby promoting the improvement of the accuracy of tea disease identification. **Figures 6**, **7** are ECA-ResNet50 and ResNet50 confusion matrices, respectively.

**Table 3:** Ablation experiments.

Join ECA	Replace the 3×3 convolution kernel	Accuracy%	Precision%	Recall%	F1%
×	×	89.88	89.90	89.88	88.89
√	×	92.5	92.61	92.5	92.55
×	√	90.68	90.69	90.69	90.69
√	√	93.06	93.09	93.06	93.07

× is not added, √ is added.

### 3.5 Comparative experiments with other datasets

To ascertain the versatility and generalizability of the model introduced in this research, extending beyond tea disease identification, we sourced disease image exemplars of apple and corn crops from the publicly accessible PlantVillage dataset (github.com/spMohanty/PlantVillage-Dataset), and each crop contained three different disease types, including 3000 apple disease images and 3192 maize disease images. The image data is divided into 80% training set and 20% test set. The ECA-ResNet50 model was then trained and tested with the original ResNet50 model, and the outcomes, presented in **Table 4**, indicate that the ECA-ResNet50 model demonstrates exceptional performance in the recognition of apple and maize diseases, and its accuracy is significantly improved compared with the unimproved ResNet50 model, which is 9.43% higher in apple disease identification and 4.17% higher in maize disease identification. This experimental endeavor conclusively establishes that the model presented in this research transcends the confines of solely tea disease identification, but also has a wide range of applicability, and can be effectively applied to the disease detection of other crops.

**Table 4:** Comparative experiments with other datasets.

Model	Plant species	Type of disease	Precision%	Recall%	F1%	Accuracy%
ResNet50	Apple	Scab	79.60	97.50	87.65	89.39
		Black rot	97.50	99.50	98.49	
		Red Star Disease	100	71.20	83.18	
	Corn	Gray spot disease	88.00	92.50	90.19	93.55
		rust	99.60	98.70	99.15	
		Big spot disease	92.10	88.40	90.21	
ECA-ResNet50	Apple	Scab	100	96.50	98.22	98.82
		Black rot	98.00	100	98.99	
		Red Star Disease	98.50	100	99.24	
	Corn	Gray spot disease	89.50	94.50	91.94	94.65
		rust	99.60	99.60	99.60	
		Big spot disease	94.10	88.90	91.43	

### 3.6 Other models than experiments

To assess the performance of the model introduced in this research in an unbiased manner, eight classical network models, including AlexNet (Huang et al., 2024), MobileNet (Sandler et al., 2018), and VGG16 (Zhao et al., 2024), were used to test and compare on the tea disease dataset, and the specific comparison results are shown in **Table 5**. The tabular data underscores the notable superiority of the ECA-ResNet50 model in the realm of tea disease identification, and its accuracy exceeds that of AlexNet (2.68%), MobileNet (7.18%), VGG16 (1.81%), ResNet34 (2.43%), ResNet50 (3.18%) and ResNet101 (2.62%). Only slightly lower than InceptionResnetv2 model (0.57% lower) and lower than Transformer (1.43% lower). Nonetheless, it is pertinent to mention that the InceptionResnetv2 model and Transformer model exhibits a considerably higher level of complexity in comparison to ECA-ResNet50. In summary, the ECA-ResNet50 model not only performs well in tea disease identification, but also has high robustness, which is a relatively lightweight model with superior performance.

**Table 5:** Comparative experiments with other models.

Model	Accuracy%	Precision%	Recall%	F1%
AlexNet	90.38	90.75	90.38	90.56
MobileNet	85.88	85.30	85.88	85.59
VGG16	91.25	91.41	91.25	91.33
ResNet34	90.63	90.70	90.63	90.66
ResNet50	89.88	89.90	89.88	88.89
ResNet101	90.44	90.54	90.44	90.49
InceptionResnetv2	93.63	93.78	93.63	93.71
ECA-ResNet50	93.06	93.09	93.06	93.07
Transformer	94.49	94.38	94.10	94.20

## 4 Conclusion

To address the challenge posed by the difficulty in identifying tea diseases amidst the intricate backdrop of tea gardens, a tea disease identification model based on ECA attention mechanism and ResNet50 network was proposed, namely ECA-ResNet50. In this study, utilizing ResNet50 as the fundamental network structure enhances the model’s capability to discern tea disease traits within the intricate environment of tea gardens. Using three 3×3 convolutional kernels to replace the 7×7 convolutional kernels of the first layer of ResNet50, the strategy of using multi-layer small convolutional kernels can not only refine the granularity of feature extraction and improve the accuracy of disease identification, moreover, it augments the model’s learning prowess and intricacy while optimizing performance through parameter reduction and network depth enhancement. The incorporation of the ECA attention mechanism fosters the model’s ability to prioritize salient feature details within the imagery, which effectively enhanced the learning and recognition ability of tea disease characteristics and improved the overall performance of the model. Compared with the original ResNet50 model, the identification accuracy of ECA-ResNet50 on the tea disease dataset was improved by 3.18%. At the same time, its performance is also better than that of six other commonly used network models (such as AlexNet, MobileNet, VGG16, etc.). In addition, the ECA-ResNet50 model has also achieved good results in other plant datasets, which fully demonstrates the effectiveness and generalization of the model.

In this study, the tea disease identification model based on the ECA attention mechanism and ResNet50 network realized the accurate and efficient identification of seven tea diseases and one healthy leaf in the complex background of tea garden, which has certain significance for the prevention and control of tea garden diseases. However, the number of tea diseases in the dataset used in this study was relatively small, and some of the diseases were similar in color and characteristics, and may even appear in the same leaf, presenting a complex disease combination. In subsequent studies, the number of images of tea diseases will be expanded; The versatility and robustness of the model will be further improved, and the design will be lightweight to be embedded in different mobile equipment for tea gardening, thus, offering valuable insights for the intelligent oversight and management of the tea cultivation industry.

## Data Availability

The raw data supporting the conclusions of this article will be made available by the authors, without undue reservation.
